# Effect of Erythrina mulungu on anxiety during extraction of third molars

**DOI:** 10.4317/medoral.19511

**Published:** 2014-06-01

**Authors:** Maria L. Silveira-Souto, Carla R. São-Mateus, Liane M. de Almeida-Souza, Francisco C. Groppo

**Affiliations:** 1 Department of Dentistry of the Federal University of Sergipe, Aracaju, Sergipe, Brazil; 2 Department of Physiological Sciences, Piracicaba Dental School, University of Campinas,Piracicaba, São Paulo, Brazil

## Abstract

Objectives: The aim of the present study was to evaluate the effect of Erythrina mulungu on the control of dental anxiety in patients who had under gone bilateral extraction of asymptomatic, impacted mandibular third molars. Material and Methods: In a randomized, double-blind, crossover study, 30 healthy volunteers (5 men and 25 women, over 18 years of age), received either 500mg of E.mulungu (Mulungu Matusa®) or 500 mg of placebo, p.o., one hour before surgical procedure. The level ofanxiety was assessed through questionnaire sand physical parameters, such as blood pressure, heart rate andoxygen saturation. Data were analyzed by Chi-square test, ANOVA (Tukey test) and Friedman with significance level of 5%. Results: A higher preference (Chi-square, p = 0.0062) for E. mulungu was observed for both genders. Volunteers with higher anxiety levels tended to to prefer E. mulungu. No statistically significant differences were verified in blood pressure (one-way ANOVA, p = 0.1259), heart rate (Friedman, p> 0.05) and oxygen saturation (Friedman, p = 0.7664) among periods and types of treatments. Conclusions: E. mulungu showed an anxiolytic effect without significant changes in physiological parameters. It could be considered as an alternative to control the anxiety in adult patients undergoing mandibular thirdmolars surgery.

** Key words:**Anxiety, Erythrina mulungu, third molar, oral surgery.

## Introduction

From the simplest procedure to a more invasive one, such as surgery, anxiety is a common problem faced in the dentist office. When these feelings of anxiety and fear occur due to the prospect of dental treatment, it is known as dental anxiety (1,2). Fear and anxiety about dental treatment are important clinical problems and they occur in 40-50% of the general population (3). Among dental procedures, minor oral surgeries tend to cause a lot of anxiety to patients because they are linked to the possibility of pain. The surgery to remove the third molaris considered the most dreadful for patients, even higher than those surgeries performed toremove hard and soft tissue from injuries or insertion of implants (4).

The emotional and psychological states of the patient can affect treatment, impair the absorption of drugs, or even cause undesirable physiological changes (2). Thus, fears, phobias, stressful situations and depression can often alter the physiological functions of the body, reduce the excitability threshold (making the body feel more pain), alter the immune response, and have a negative impact on the trans and postoperative periods (4).

Given the fact that the dentist is routinely in contact with anxious patients, methods for controlling anxiety are used when such condition can alter the performance of a safe and quality procedure. Anxiety can be controlled by both pharmacological and non-pharmacological methods. For this, the most used method is the verbalization one, where the dentist should be able to understand, guide, soothe and comfort the patient regarding the procedures to be performed. When this is not enough, pharmacological methods are used, such as anxiolytics, which act reducing anxiety. Benzodiazepines are the most commonly used anxiolytics (5). However, they have important side effects. Patients using benzodiazepines can not go unaccompanied to a consultation, drive or operate machinery during treatment.

Because of the difficulties with the use of this group of drugs, one may resort to the use of herbal medicines. These are substances obtained from plants, which can be used as handmade medicines in the form of teas, solutions orpills (6).

*Erythrina mulungu*, a Southern Brazilian native plant which produces alkaloids and steroids, is a herbal medicine known for its good control of anxiety. Popularly known asmulungu (7), *Erythrina mulungu* is a medium-sized tree found in tropical regions and itsbark and seed have been used in folk medicine due to their anticonvulsant, analgesic, sedative, hypnotic and hypotensive properties (8).

Studies in mice and rats show that water-alcoholic extracts of E. mulungu are central nervous system depressants, they alter the responses related to anxiety, but they do not affect motor coordination (6-8). Although the benefits of anxiolytic effects of the *E. mulungu* can be applied to the dental clinic for anxiety reduction without presenting the constraints of benzodiazepines, there is a dearth of research on the use of this plant for the control of dental anxiety. Thus, this study aimed to assess the efficacy of *E. mulungu* in controlling anxiety during dental procedure.

## Material and Methods

The study was submitted for review and approval to the University Hospital Research Ethics Committee of the Federal University of Sergipe by the protocol CEP 401/2011 and CAAE number 0366.0.107.000-11,in a meeting held on 07/12/2011, and consort number NCT01948622. In this research, characterized as a randomized, double-blind, crossover study, 30 volunteered patients from the Department of Dentistry of the Federal University of Sergipe (DOD/UFS) were selected, after diagnosis and indication of bilateral extraction of asymptomatic, impacted mandibular third molars, according to Pell and Gregory classification used by Almendros-Marqués, Berini-Aytés & Gay-Escoda (9). This method classifies mandibular third molars into 9 different categories based on their vertical position (relative to the cemento enamel junction and the occlusal plane) and their horizontal position (relative to the ascending mandibular ramus).

All participants were informed of both risks and benefits of the study, and signed an informed consent. Exclusion criteria were: patients under the age of 18; any general health problem based on the medical history and physical examination; history of use of any medication within 15 days before the beginning of the research; history of hypersensitivity to drugs, substances or materials used in this experiment; pregnancy or lactation; history of pericoronitis.

The study participants randomly received either Mulungu Matusa® 500 mg (two capsules of 250 mg each) or placebo (starch, two capsules), orally one hour before the start of surgical procedures, at either the first or second intervention (right or left side). It is noteworthy that both the placebo and the capsule of *Erytrina mulungu* were absolutely equal in size and shape. The drug was given to the patient by the first researcher, coded as “Protocol 1” or “Protocol 2”. Both protocols were only identified at the end of the experiment. Randomization was done with Random Number Generator Pro 2.15 software and it was established that 15 patients received “Protocol 1” and 15 patients received “Protocol 2” in the first surgery. Therefore, everyone involved in the research, volunteers, surgeon and researcher, had no prior knowledge of pharmacological treatments that were being used (double-blind study).

In order to avoid the pain and swelling after surgery, a single dose of intramuscular dexamethasone (8mg), 30 minutes before surgery, was administered. Before the surgery, oral antisepsis was performed by vigorous rinsing, for one minute, with an aqueous solution of chlorhexidinedigluconate (0.12%). In the extra-oral antiseptisis, an alcoholic solution of polyvinylpyrrolidoneiodine (PVP-I) 10% was used.

Local anesthesia was performed using the Vazirani-Akinosi mandibular nerve block technique, according to Haas (10), which consists of positioning the needle tip in the pterygomandibular space. For this blockade, one cartridge (1.8 ml) of 2% lidocaine with 1:100,000 of epinephrine was used. The needle was inserted into the tissue in the distobuccal vestibule opposite the second or third mandibular molar just medial to the coronoid notch until bone was contacted and 0.25 mL of articaine (4%) with 1:100,000 of epinephrine was released, only then the buccal nerve was anesthetized, according to Reed *et al.* (11).

Surgical procedures occurred in two sessions, one for each side of the hemi-jaw. Extractions of impacted mandibular third molars were done by the operator, a maxillofacial surgeon from the DOD/UFS. The surgical technique used was performed according to Jansma *et al.* (12).

Patients were instructed for local hemostatic care, feeding, cleaning the operated region, restriction of physical exertion, and other routine recommendations usually given in this type of intervention. In the following day of each tooth extraction, a local application of aqueous chlorhexidinedigluconate 0.12% for the dental plaque control, every 12 hours for 7 days, was recommended. The suture was removed on the seventh day. The volunteers also received three tablets of 750 mg paracetamol, as analgesic medica-tion, being advised to take one tablet every 6 hours only if pain. The minimum interval between the first and second surgery was 15 days.

The assessment of the subjects’ anxiety level was conducted through questionnaires and physical parameters, and it was divided into three phases: Phase I (baseline), Phase II (day of surgery) and Phase III (return visit).

-Phase I - baseline: during the initial consultation, a week before the day scheduled for the first intervention,the Modified Corah Dental Anxiety Scale was used. The scaleconsists of a questionnaire with four questions, each with five possible answers, which evaluates the feelings, signs and reactions of patients related to dental treatment. Each alternative response received a certain score (1-5), and, ultimately, the patients were classified according to their level of anxiety based on the sum of these points as: very little anxious, mildly anxious, moderately anxious and very anxious. The terminology used in the questions was adapted to the needs of the research.

-Phase II -the day of surgery: the level of anxiety was assessed by the second researcher and the operator responsible for the surgery, who answered questions of the same questionnaire at the end of each surgery. In this questionnaire,both the researcher and operator classified the patient as quiet, moderately anxious or very anxious and, in the case of patients moderately or very anxious, it was necessary to answer in which surgical time this happened (at the moment he/she entered the operating room, during antisepsis, anesthesia, surgery itself or in end of the procedure). In addition to responding the questionnaire, the second researcher tabulated data from the evaluation of blood pressure (mmHg), heart rate (bpm) and the level of oxygen saturation (SpO2) before drug administration, 30 minutes after drug administration and during the following surgical times: local anesthesia, incision, tooth removal and suturing.

-Phase III - return visit: after each surgical procedure, the volunteers received a self assessment form, to be answered in the days following the surgery. They were supposed to answer if they were tranquil, moderately anxious or very anxious. And also if they rememberede very thing from the surgery, the majority of events,some particular time, almost nothing or nothing at all, with the purpose of expressing the presence or absence of anterograde amnesia. The presence of possible side effects from the medication used in this study was also questioned. Upon completion of the second surgery, they answered which procedure they preferred, if the first or the second surgery.

After data collection, they were tabulated and analyzed by the following statistical tests: chi-square, t-test, ANOVA and Tukey test, Friedman, Fisher’s exact test with significance level of 5%.

## Results

**Figure 1:**
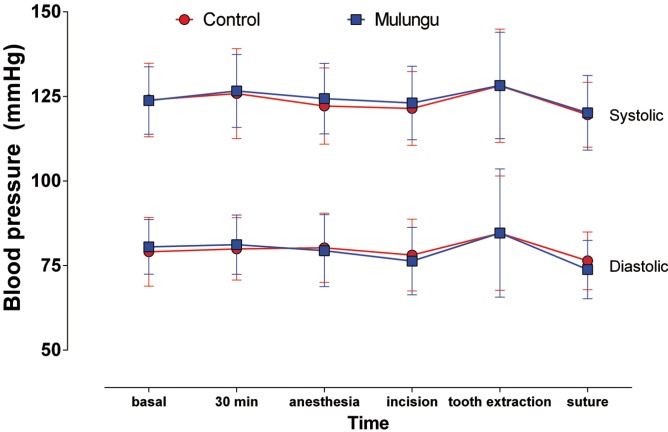
Mean (standard deviation) of systolic and diastolic blood pressure.

There were 30 volunteers, 25 females with mean age (standard deviation) of 22.4 (3.6) years; and 5 males with mean age of 22.6 (4.0) years. There were no statistically significant age differences (t test, p = 0.9296) among genders (Fig. 1).

**Figure 2:**
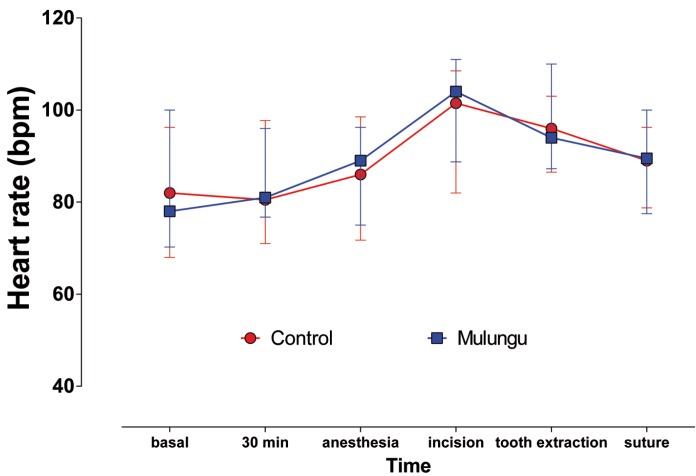
Median (interquartile range) of the heart rate.

Charts from 1 to 3 show, respectively, the measures of blood pressure, heart rate and SpO2. There were no statistically significant differences (one-way ANOVA, *p* = 0.1259) neither among groups nor among the times, regarding the systolic pressure. Diastolic blood pressure did not show significant statistical differences among groups, but there was a significant increase (ANOVA, Tukey, *p* <0.05) during tooth removal in both groups (Fig. 2).

**Figure 3:**
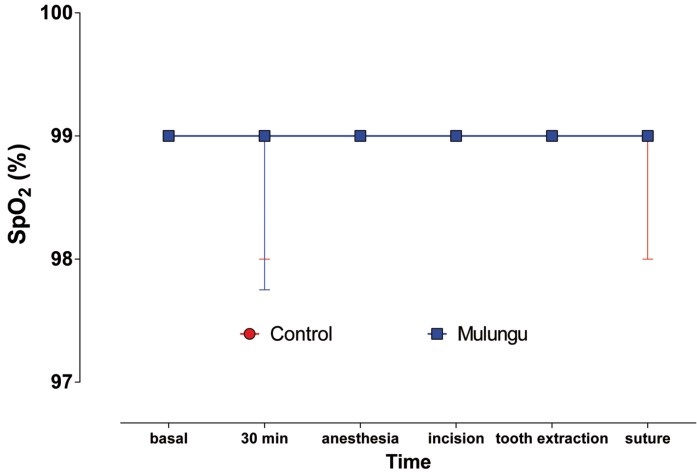
Median (interquartile range) of SpO2.

Considering the heart rate, it was observed that there was no significant statistical differences among groups (Friedman, *p*> 0.05), but there was significant statistical differences among the times studied. Thus, there was a significant increase in heart rate during the incision, which remained elevated until the suture was done for both groups (Fig. 3).

There was also no significant statistical differences (Friedman, *p* = 0.7664) among groups or times regarding SpO2.

 Table 1 shows the distribution of patients regarding the anxiety level perceived by the operator, the researcher and the patient. There was no distribution among the perception of the operator and the researcher, therefore the data were pooled. It could be observed that there were no statistically significant differences (Fisher’s exact test, *p*> 0.05) among the relative distributions observed in each group.

**Table 1:**
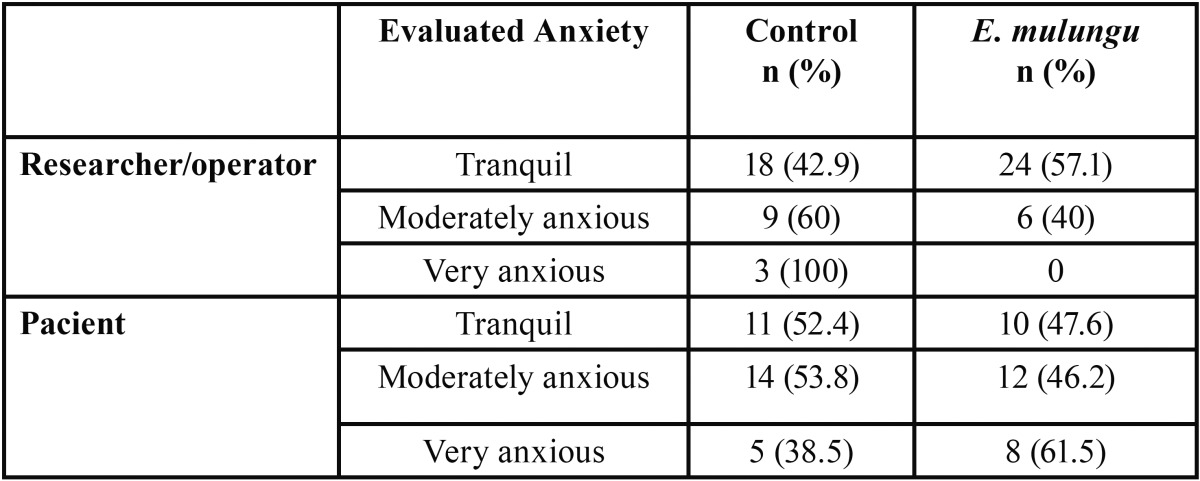
Anxiety level perceived by the operator, the researcher and the patient.

 Table 2 shows the relative distribution of perceived anxiety assessed by the operator/researcher and the patient. As it can be observed in the refered table, the perception of the patient’s anxiety by the operator/researcher was the same as the patient in 50% of cases.

**Table 2:**
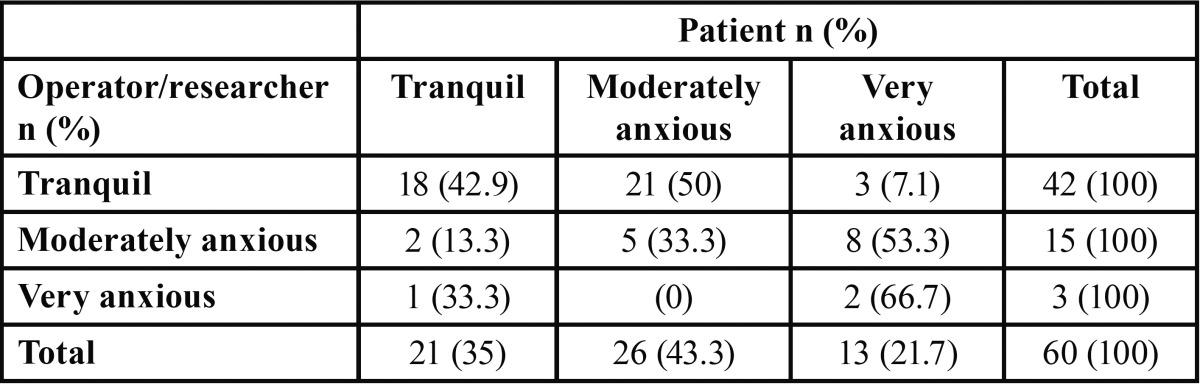
Distribution of perceived anxiety assessed by the operator / researcher and the patient.

 Table 3 shows the surgery moment when patients reported anxiety. There were no differences between the operative times reported by patients as a point of anxiety for both groups.

**Table 3:**
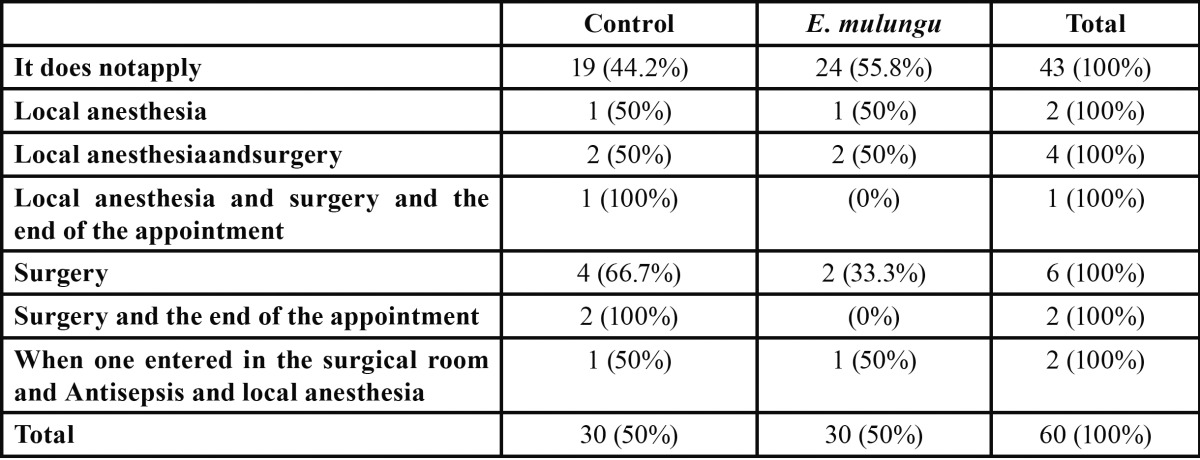
Surgery moment when patients reported anxiety.

 Table 4 shows the preference either for *E. mulungu* or the placebo by gender or by their stated anxiety level. In general, we observed a greater preference (Chi-square, *p* = 0.0062) for *E. mulungu* in both genders. Besides that, the higher the anxiety level, the greater the tendency to prefer *E. mulungu*.

**Table 4:**
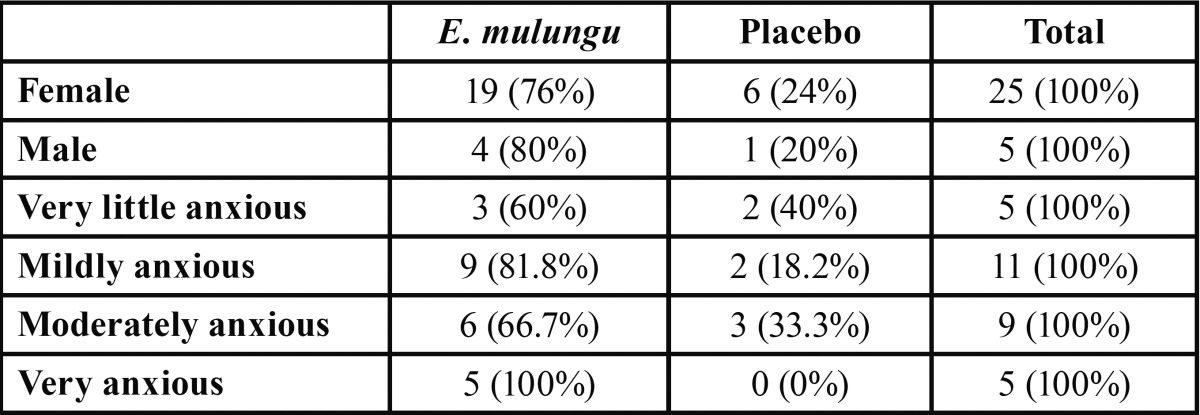
Preference for E. mulungu or placebo by gender of individuals or of their stated anxiety level.

## Discussion

Minor oral surgery under local anesthesia is a common procedure and has a relatively short period of recovery, but its physical and psychological impacts make it a stressful experience (13). Patients undergoing oral maxillofacial surgery showed higher levels of anxiety compared to those who underwent other types of surgery, such as gastrointestinal disorders (14). The anxiety control in dental surgical procedures proves to be of great value, as the level of anxiety can alter the postoperative comfort of the patient. Thus, preoperative anxiety is an accurate indicator of postoperative pain and recovery after oral surgery (15).

The anxiety level of the subjects who participated in this study during dental treatment was measured by the Modified Corah Dental Anxiety Scale. This scale has been used in several studies (16-20) for its simplicity to be applied, for allowing adaptation in the translation of the questions and answers of the questionnaire to the native language and for the fact that it shows validity and reliability for obtaining results.

The present study evaluated the anxiolytic effect of *E. mulungu* for surgical removal of mandibular third molars. *E. mulungu* has been used in herbal compositions and medicines in Brazil and in the United States for its actions in the central nervous system (21,22). We chose to use the *E. mulungu* due to the fact that its extracts are already used in Brazilian folk medicine because of its anticonvulsant, antidepressant, analgesic, sedative, hypnotic, and hypotensive effects (21,22).

The anxiety level of patients and the effectiveness of the treatments were evaluated by the researcher and by the surgeon on the day of the intervention (Phase II). In the perspective of these observers, the results indicate that patients appeared to be more relaxed when the protocol used was the one with *E. mulungu*. Furthermore, the uniformity of opinion between the researcher and operator can validate the method of the applied evaluation, since both did not know what protocol was being used at the time of surgery. Subsequently, the patients were asked about which surgery they felt more comfortable with during the Phase III of the research. Most patients reported a preference for protocol with *E. mulungu*. It was also observed that the higher the level of anxiety, according to the Modified Corah Dental Anxiety Scale, the higher the preference for protocol with *E. mulungu*.

The high preference for protocol with *E. mulungu* can be explained by the anxiolytic effects found in the extracts of this plant in animals conducted studies. Studies in rats and monkeys have shown that acute or chronic doses of *E. mulungu* have anxiolytic effects (6,7,21,23). Yet, researches indicate that the anxiolytic effect of *E. mulungu* is not associated with motor abnormalities (6,21,23). None of the volunteers involved in the study showed motor impairment, and drowsiness was the only side effect re-ported by some patients during the protocol with *E. mulungu*. The permanence of motor reflexes can be considered a major advantage of the use of this natural anxiolytic in comparison with the anxiolytic commonly employed in dentistry, such as benzodiazepines, that cause motor disorders and thus require that patients come accompanied to the surgical procedure.

No differences were found among the values of blood pressure, heart rate and oxygen concentration between the two groups evaluated in this study. However, there are reports that *E. mulungu* presents hypotensive effect (21). The maintenance of the oxygen concentration may also be considered an advantage of *E. mulungu* compared to benzodiazepines, which can cause respiratory depression.

Studies conducted in monkeys with water-alcoholicextracts of *E. mulungu* indicate the presence of anti-contraceptives effects, because of an action on the spinal level, which explains the popular use of this plant in order to obtain analgesic effects (8). This analgesic property can influence in a more comfortable postoperative period after dental surgical procedures.

The use of *E. mulungu* provided a quieter surgical procedure for most patients. As it was mentioned on the results, the higher the level of the patient’s anxiety, the higher the preference for the protocol with *E. mulungu*. Therefore, the use of this natural anxiolytic in anxious patients may be advantageous, besides the fact that they do not cause respiratory depression and motor abnormalities. However, as there is a lack of studies in humans, it is not possible to confirm the results found. It is important to confirm results, so that we can be able to test optimal dosages and to refine research models to assess clinically the anxiety during dental treatment and its pharmacological control.
